# Complete Genome Sequence of Human Respiratory Syncytial Virus from Shanghai, China

**DOI:** 10.1128/genomeA.00989-15

**Published:** 2015-09-10

**Authors:** Xuemin Fu, Yanwei Cheng, Zhixiang He, Wei Dong, Ke Lan, Chiyu Zhang, Yihong Hu

**Affiliations:** a Pathogen Diagnostic Center, Institut Pasteur of Shanghai, Chinese Academy of Sciences, Shanghai, China; b Life Science College, Luoyang Normal University, Luoyang, China; c Pediatric Department, Shanghai Nanxiang Hospital, Shanghai, Jiading District, China

## Abstract

We report here the complete genome sequence of human respiratory syncytial virus isolated from an outpatient child with fever and respiratory symptoms in Shanghai, China, in 2014. Phylogenetic analysis showed that the full-length respiratory syncytial virus (RSV) genome sequence belongs to human RSV (HRSV) group A.

## GENOME ANNOUNCEMENT

Human respiratory syncytial virus (HRSV) of the genus *Pneumovirus* is one of the most common pathogens causing severe acute lower respiratory tract infections (ARTIs), accounting for 15 to 40% of pneumonia/bronchiolitis cases in children (1). It is a nonsegmented negative-sense single-strand RNA virus. The genome of HRSV is about 15 kb, which encodes eight structural proteins, including nucleocapsid protein (N), matrix protein (M), phosphor protein (P), glycoprotein (G), fusion protein (F), large protein (L), two virus integral membrane proteins (M2-1 and M2-2), two nonstructural proteins (NS1 and NS2), and a currently unknown protein (SH). According to antigenic and genetic variability in the G gene, HRSV is categorized into two groups (A and B). Groups A and B were further subdivided into 11 (ON1, GA1 to GA7, SAA1, NA1, and NA2) and 20 (GB1 to GB4, BA1 to BA10, SAB1 to SAB4, URU1, and URU2) genotypes (2, 3), respectively. The HRSV complete genomic sequence information will benefit the molecular epidemiological investigation of RSV and vaccine development.

In this case, we obtained a clinical nasopharyngeal swab, which was collected from an outpatient child with fever and respiratory symptoms in Shanghai Nanxiang Hospital, Shanghai, China, in 2014. The sample was immediately transported to our laboratory in virus transport medium, and total RNA was extracted from 140 µl of the sample using a QIAamp viral RNA minikit (Qiagen, Germany) (4). The RNA was reverse transcribed into cDNA and then was tested using a modified multiplex PCR. This reverse transcription PCR assay (RT-PCR) was developed to detect 17 kinds of respiratory viruses (including HRSV) simultaneously. The HRSV-positive sample was chosen (4) and amplified to obtain the complete genome sequence. Briefly, 14 overlapping genomic segments were amplified to cover about 15 kb of the full-length genome of RSV using RT-PCR. The primers used in the amplification were described previously (5), and additional primers were designed as necessary (forward primer, 5′-CAACATCTCACCATGCAAGCCA-3′; reverse primer, 5′-TGAGGATTGGCAACTCCATTGTTA-3′). The purified PCR amplification products were T-A cloned into pMD18-T vector (TaKaRa Biotechnology Co., Ltd.). The recombinant plasmid containing HRSV genomic fragments was directly sequenced by a commercial sequencing service company with the positive reverse bidirectional determination sequencing method.

The sequenced DNA fragments were assembled into complete genomes by Vector NTI (version 11.5). The whole-genome sequence of strain HRSV/B614122905 was 15,264 nucleotides (nt) long. The contents of A, U, G, and C were 38.8%, 27.7%, 15.8%, and 17.7%, respectively. The sequence was aligned with other HRSV genomic sequences retrieved from GenBank, and a phylogenetic tree was constructed using the neighbor-joining method with MEGA 5.0. Phylogenetic analysis showed that strain HRSV/B614122905 belongs to genotype ON1 of the RSV A group. This study suggests the necessity of continuous monitoring of genetic variation of more current HRSV strains and provides a public health warning for possible outbreaks in the future. The increasing frequency of the detection of HRSV strains with the ON1 genotype worldwide indicates that this genotype may have great advantages for escaping current host immunity (6).

### Nucleotide sequence accession number. 

The whole-genome sequence of strain HRSV/B614122905 has been deposited in GenBank under the accession no. KT285064.
